# Decellularized Green and Brown Macroalgae as Cellulose Matrices for Tissue Engineering

**DOI:** 10.3390/jfb15120390

**Published:** 2024-12-23

**Authors:** Caitlin Berry-Kilgour, Indrawati Oey, Jaydee Cabral, Georgina Dowd, Lyn Wise

**Affiliations:** 1 Department of Pharmacology and Toxicology, School of Biomedical Sciences, University of Otago, Dunedin 9054, New Zealand; caitlin.berrykilgour@postgrad.otago.ac.nz; 2 Department of Food Sciences, University of Otago, Dunedin 9054, New Zealand; indrawati.oey@otago.ac.nz; 3 Department of Microbiology and Immunology, School of Biomedical Sciences, University of Otago, Dunedin 9054, New Zealand; jaydee.cabral@otago.ac.nz; 4 The New Zealand Institute for Plant and Food Research Limited, Nelson 7043, New Zealand; georgina.dowd@plantandfood.co.nz

**Keywords:** macroalgae, seaweed, cellulose, decellularization, matrix, scaffold, fibroblast, skin, tissue engineering

## Abstract

Scaffolds resembling the extracellular matrix (ECM) provide structural support for cells in the engineering of tissue constructs. Various material sources and fabrication techniques have been employed in scaffold production. Cellulose-based matrices are of interest due to their abundant supply, hydrophilicity, mechanical strength, and biological inertness. Terrestrial and marine plants offer diverse morphologies that can replicate the ECM of various tissues and be isolated through decellularization protocols. In this study, three marine macroalgae species—namely *Durvillaea poha*, *Ulva lactuca*, and *Ecklonia radiata*—were selected for their morphological variation. Low-intensity, chemical treatments were developed for each species to maintain native cellulose structures within the matrices while facilitating the clearance of DNA and pigment. Scaffolds generated from each seaweed species were non-toxic for human dermal fibroblasts but only the fibrous inner layer of those derived from *E. radiata* supported cell attachment and maturation over the seven days of culture. These findings demonstrate the potential of *E. radiata*-derived cellulose scaffolds for skin tissue engineering and highlight the influence of macroalgae ECM structures on decellularization efficiency, cellulose matrix properties, and scaffold utility.

## 1. Introduction

Engineering of biological tissues requires a multifaceted approach providing the correct cell types, structural support, and biological cues [1]. Structural support in the form of scaffolding is critical in providing a three-dimensional (3D) matrix for cell attachment and proliferation [1,2]. These scaffolds are composed of synthetic or naturally derived biomaterials that intrinsically, or through fabrication, replicate the extracellular matrix (ECM) of the desired tissue [3]. Bottom-up approaches to scaffold fabrication, such as 3D printing and electrospinning [4,5], use materials extracted from natural or chemical sources to synthesize a matrix. Meanwhile, top-down approaches, such as decellularization, isolate the tissue ECM using chemical, enzymatic, or physical means [6]. Tissue engineering scaffolds have been generated through the decellularization then recellularization of human and animal tissues, including the dermis [7,8], vasculature [9,10], whole lung [11,12], and whole heart [13,14]. The advantage of this approach is that the ECM retained within the decellularized scaffold intrinsically meets the functional requirements of the tissue being generated [6]. However, there are obstacles to the widespread use of mammalian ECM as scaffolds, including immunogenicity [15,16], donor-specific limitations to recellularization [17,18], and ethical or religious barriers [19].

Plants are emerging as a source of tissue engineering materials and scaffolds. The main structural element of plant ECM is cellulose, a key constituent within the cell wall that provides strength and rigidity [20]. This polymer has an organized structure comprising hydrophilic glucose monomers, the degradation of which requires specific enzymes that are lacking in mammals. Cellulose, therefore, offers advantages for tissue engineering in that it provides a moist environment, positively charged surfaces for cell interactions, and stability with minimal deformation over a long period of time. Cellulose fibers extracted from bacterial cultures, wood pulp, cotton, and flax, including those processed into nanofibers and nanocrystals, have been studied in the form of hydrogels, films, and dressings for tissue engineering applications with promising results [21,22,23]. However, extraction results in loss of the plant ECM structure, which can mimic the hierarchal architecture of mammalian ECM to control cell fate and guide tissue formation [24,25]. This limitation has been addressed through decellularization methods that enable 3D cellulose matrices to be isolated while retaining native ECM structures. For example, decellularized matrices derived from terrestrial plants have been utilized for in vitro engineering of adipose tissue (apple) [25], cardiac tissue (spinach) [26], bone (apple, bamboo stem, and carrot) [27,28], tendon (celery) [25], skeletal muscle (apple and onion) [29,30,31], skin (apple) [25,31,32], and vasculature (spinach and parsley) [32,33]. In most examples, cellulose-based matrices were prepared using one- to two-step protocols involving ionic or non-ionic surfactants to lyse the plant cell wall and remove cellular content. Further, the recellularization of decellularized apple scaffolds has been shown in vivo, four to eight weeks after subcutaneous implantation in murine recipients [30,34].

Terrestrial plants have been the primary source of decellularized cellulose-based matrices but interest is increasing in marine plants for this purpose. Macroalgae are harvested from coastal waters and farmed in large aquaculture enterprises for food, fuel, and biomedical applications, with many used as sources of polysaccharides for wound healing, tissue engineering, and drug delivery [35,36]. Cellulose from macroalgae has not been explored to the same extent but offers a higher degree of crystallinity, biocompatibility, and stability than terrestrial plant-derived cellulose [37,38]. Macroalgae are classified into *Chlorophyta* (green), *Phaeophyceae* (brown), and *Rhodophyta* (red) species that differ greatly in morphology, with thalli shaped as cylinders, blades, or sheets, composed of epidermal, cortex, and/or medulla layers of cells that vary in structure [39]. Intriguingly, the multilayered architecture of macroalgae resembles that of human tissues such as the skin, with matrices that mimic ECM basement membranes and interstitial fibers [40]. As the cell wall of macroalgae contains polysaccharides, with little to no lignin in comparison to terrestrial plants [37,38], this cellulose-based ECM matrix can be readily isolated through decellularization. The first decellularization study of green microalgae used a four-step chemical and thermal treatment protocol to bleach pigments and remove DNA, proteins, lipids, hemicellulose, and polysaccharides from the cellulose matrices of *Ulva* sp. and *Cladophora* sp. [41]. The resulting scaffolds differed in ECM structure and utility, with the fibrous and porous matrices from *Cladophora* sp. and *Ulva* sp., respectively, varying in support of fibroblast culture. This led to the current investigation, which is aimed at understanding the impact of native cellulose ECM structure on decellularization efficiency and scaffold utility. Two brown macroalgae, *Durvillaea poha* and *Ecklonia radiata*, and the green macroalgae *Ulva lactuca*, were selected based on varied thallus morphology. Decellularization protocols were trialed to retain the distinct cellulose ECM matrix for each species while maximizing pigment and DNA clearance and minimizing chemical and heat usage. The suitability of each cellulose-based matrix as a scaffold for the culture of human dermal fibroblasts was then evaluated.

## 2. Materials and Methods

### 2.1. Macroalgae Sources

*Ecklonia radiata* was supplied by AgriSea New Zealand Seaweed Limited (Paeroa, New Zealand). Whole beach cast *E. radiata* collected from the east coast of the North Island of New Zealand was air-dried, then transported and stored in cloth sacks at room temperature, with the secondary laminae thoroughly washed then rehydrated in distilled water for 10 min prior to processing.

Samples of *U. lactuca* and *D. poha* were collected from the intertidal zone at St Kilda Beach and Brighton Beach (Otago, New Zealand), respectively. The samples were cut cleanly, keeping the holdfast intact to minimize impact on growth in accordance with sustainable harvest practices [42]. The *D. poha* blade, palm, and stipe were separated, then along with the *U. lactuca* thalli, were transported in sea water in an airtight container. Whole *U. lactuca* thalli and *D. poha* medulla samples were cut into pieces weighing approximately 10 g, then either processed immediately to assess their native structure, pigment, and DNA content or wrapped in tin foil and stored at −20 °C under airtight conditions, prior to chemical treatment.

### 2.2. Histological Analysis of Macroalgae Structure

Fresh or rehydrated macroalgae samples (approximately 1 cm^2^) were fixed in 10% neutral buffered formaldehyde for 48 h and then dehydrated in 70% ethanol for 24 h before embedding in paraffin wax (all Thermo Fisher Scientific, Waltham, MA, USA). The samples were cut into 6 µm sections using a rotary microtome. Sections were dried, deparaffinized, and rehydrated prior to staining with haematoxylin and eosin (H&E) (Leica Biosystems, Surgipath, IL, USA) in order to visualize the native seaweed structure [31]. Haematoxylin stains nuclei a purple–blue color, while basic intracellular and extracellular molecules such as proteins are stained pink. After staining, sections were mounted using DPX mounting medium (Sigma-Aldrich, Saint Louis, MO, USA), then imaged under white light using the Olympus BX51 microscope, DP71 digital camera, and CellSens V4.2 software (Olympus Corporation, Tokyo, Japan).

### 2.3. Scanning Electron Microscopy of Macroalgae Structure

Fresh or rehydrated macroalgae samples (approximately 5 mm^2^) were fixed in 2.5% glutaraldehyde (in 0.1 M sodium cacodylate buffer, pH 7.2) (both Sigma-Aldrich) for 4 h. Chemical processing was then undertaken in a Lynxel Tissue Processor (Australian Biomedical Corporation Ltd., Mount Waverley, Australia) at room temperature. The samples were washed three times (20 min each) in 0.1 M sodium cacodylate buffer before being transferred to 1% osmium tetroxide (in water) (Sigma-Aldrich) for 1 h. Samples were again washed three times (20 min each) in 0.1 M sodium cacodylate buffer before undergoing serial dehydration in ethanol (20 min each at 20%, 40%, 60%, 80%, 90%, 95%, 100% (twice), then a final hour at 100%). Samples were critical point dried in a Bal-Tec CPD-030 critical point dryer (Bal-Tec AG, Balzers, Liechtenstein) with liquid CO_2_ and absolute ethanol as the intermediate fluid. When dried, the samples were cut down to an appropriate size and mounted on aluminium stubs using double-sided carbon tape. All samples were then coated with approximately 10 nm of gold/palladium (80:20) using a Quorum Q150V ES PLUS coater (Quorum Technologies, East Sussex, UK) before being imaged in a Zeiss Sigma 300VP field emission scanning electron microscope (SEM) operating at high vacuum, with an accelerating voltage of 5 kV. Each sample was imaged at a range of magnifications, with cross-sections, and where applicable, internal and/or external views were obtained for each sample.

### 2.4. Chemical Decellularization of Macroalgae

Defrosted or rehydrated seaweed samples were washed with distilled water to remove debris before dissection into 1 cm^2^ pieces with a sterile scalpel. To optimize the decellularization protocol, methods from the literature were selected and tested, with the primary readout being loss of pigmentation [43]. The protocol was first optimized for *D. poha* given the size and density compared to *E. radiata* and *U. lactuca*. The *D. poha* medulla samples, originating from the blade, palm, and stipe, were treated with sodium dodecyl sulfate (0.1–0.5% SDS) (Thermo Fisher Scientific) at room temperature (20 °C), 3% NaCO_3_ (Merck, Rahway, NJ, USA)/0.3% NaClO (Chemical Solutions Limited, Christchurch, New Zealand) at 60 °C, or 10% SDS with a subsequent treatment of 1% Triton X-100 (Sigma-Aldrich)/0.1% NaClO at room temperature [31,34,43] (Supplementary Materials: Table S1). All treatment steps were conducted in 20 mL volumes, in 25 mL tubes on a rocker, with weekly replenishment. Samples were assessed for pigment clearance (as described below) at set time points, or when ‘cleared’ or transparent [43]. Once the protocol for *D. poha* was developed, this was tested on *E. radiata* and *U. lactuca* samples, originating from the secondary lamina and thallus, respectively, with modifications made to reduce chemical exposure over time.

The final protocol for *D. poha* involved incubation of samples in 10% SDS (20 mL) for 5 days at room temperature. The samples were removed from this solution, then incubated in 1% Triton X-100/0.1% NaClO for 14 days at room temperature. The final protocol for *E. radiata* and *U. lactuca* involved incubation of samples in 1% Triton X-100/0.1% NaClO for 14 days at room temperature.

### 2.5. Measurement of Pigment Clearance

Loss of coloration was used as a proxy for loss of pigmentation [43]. Samples were photographed on a white tray both before (native) and after chemical treatment (treated). The pigment content was quantified from three replicate experiments using ImageJ V1.54j software (National Institute of Health, Bethesda, MA, USA). Images were converted to 8-bit and the mean gray value (MGV) of each section and the adjacent tray (background) was measured within a uniform rectangular area. Pigment content was calculated by subtracting the background value from that of the sample:

$$Pigment\;content=MGV\;\left(sample\right)-MGV\;(background)\;$$

Pigment clearance in treated samples was then calculated as a percentage of that in native seaweed (prior to treatment):

$$Pigment\;clearance\;\left(\%\right)\;=\;\frac{pigment\;content\;(native)\;-\;pigment\;content\left(treated\right)}{pigment\;content\;\left(native\right)}\times 100$$

### 2.6. Measurement of DNA Clearance

Native and treated samples were frozen at −80 °C, then ground with liquid nitrogen using a mortar and pestle until a fine powder was produced. DNA was extracted using the DNeasy Plant Pro Kit (Qiagen Sciences, Germantown, MD, USA) with the following modifications. Ground sample (150 mg) was placed in the supplied tissue disruption tube, then lysed using the provided CD1 and PS solutions, according to the manufacturer’s instructions. To enhance extraction yields, TRIzol™ reagent (1 mL, Thermo Fisher Scientific) was added to the lysis mixture, with incubation for 10 min, before the addition of chloroform (Sigma-Aldrich, 400 µL), agitation, and incubation for 3 min. To isolate the chloroform fraction, samples were centrifuged at 13,000 rpm for 20 min at 4 °C. Ethanol (1 mL) was added to the chloroform fraction and this mixture was passed through the MB spin column provided in the kit. Washing and DNA elution was performed according to the manufacturer’s instructions, using the provided AW1, AW2, and elution buffers. The DNA concentration was quantified in three independent samples per species (matched native and treated), using the DS-11 FX+ fluorometer spectrophotometer (DeNovix, Wilmington, DE, USA). The DNA content was calculated relative to the initial sample weight:

$$DNA\;content\;(ng/mg)=\frac{DNA\;concentration\;(ng/µL)\;\times eluted\;volume\left(µL\right)}{sample\;weight\;(mg)\;}$$

DNA clearance in treated samples was then calculated as a percentage of that in native seaweed:

$$DNA\;clearance\left(\%\right)=\frac{DNA\;content\;\;(native)-DNA\;content\;(treated)\;}{DNA\;content\;(native)\;}\times 100$$

### 2.7. Visualization of Matrix Structure

Treated samples were processed for histological and SEM analyses, with imaging performed as described for the native macroalgae samples.

To visualize the cellulose fibers, sections were stained with calcofluor white (CW, Sigma-Aldrich), and where indicated, wheat germ agglutinin (WGA)–AlexaFluor^TM^ 594 conjugate (Thermo Fisher Scientific) was added concurrently to visualize glycans [44]. Paraffin-embedded sections were dried, deparaffinized, and rehydrated. One drop of the CW stain (undiluted) was added to the slide, with or without WGA conjugate (5.0 μg/mL in 50 µL phosphate buffered saline (PBS, Oxoid, Basingstoke, UK)). After 10 min, a coverslip was placed on the slide and the stained section was imaged using the Olympus BX51 microscope, DP71 digital camera, and CellSens software. To visualize WGA, excitation and emission wavelengths of 587 and 603 nm were used; to visualize CW, excitation and emission wavelengths of 380 and 475 nm were used.

To assess matrix porosity, the diameter of pores within the external layers of *U. lactuca* and *E. radiata* as well as gaps between the fibers in the inner layers of *D. poha* and *E. radiata* were measured in SEM images using ImageJ software. After setting the scale, the freehand line tool was used to measure the diameter of two pores or gaps (at their greatest width) per image, from three images per section and three sections per matrix. The mean diameter for each matrix was calculated from these technical replicates, with three independent matrices assessed per species.

### 2.8. Measurement of Matrix Hydration

Discs were prepared from treated samples using a sterile biopsy punch (4 mm). The discs were then freeze-dried for 24 to 48 h in a Virtis SP Scientific BenchTop Pro freeze-drier (Scientific Products, Warminster, PA, USA). To assess their swelling characteristics under mammalian cell culture conditions, the discs were weighed using a microbalance (dry weight), sterilized with 70% ethanol (1 mL) for 30 min, then solubilized in Dulbecco’s Modified Eagle Medium (DMEM) (1 mL) with 10% fetal calf serum (FCS) (both Thermo Fisher Scientific) in a 24-well tissue culture plate (Sigma-Aldrich). Samples were then incubated at 37 °C, in 5% CO_2_, for 24 h. The solubilized discs were then re-weighed (wet weight). Three replicate experiments were conducted, each with three technical replicates, for each seaweed species. Swelling was then calculated as the ratio of the wet and dry weights:

$$Swelling\;ratio=\frac{Wet\;weight\;(24\;hour)\;}{Dry\;weight\;(0\;hour)\;}$$

### 2.9. Measurement of Matrix Stability

Discs were prepared from treated samples (4 mm), sterilized, solubilized, then incubated for 7 days as described above. To assess the stability of the matrices [45], the wet discs were weighed on days 0 and 7. Three replicate experiments were conducted, each with three technical replicates, for each seaweed species. Mass loss was then calculated as the percentage of the original mass:

$$Mass\;loss\left(\%\right)=\frac{wet\;weight\left(day\;0\right)-wet\;weight\;(day\;7)\;}{wet\;weight\;(day\;0)\;}\times 100$$

### 2.10. Fibroblast Culture on Matrices

Immortalized dermal fibroblasts (BJ/5Ta, ATCC, Manassas, VA, USA) were cultured in T-175 tissue culture flasks (Sigma-Aldrich), with populations maintained between 8 × 10^3^ and 1 × 10^4^ cells per cm^2^ as per the supplier’s instructions. Cells were cultured in BJ/5Ta media containing DMEM–Medium 199 (1:4), 10% FCS, and 0.02% hygromycin B (all from Thermo Fisher Scientific), with incubation at 37 °C in 5% CO_2_.

Discs (4 mm) were prepared from treated samples, sterilized, solubilized, then air-dried in a biosafety cabinet for 10 min. Fibroblasts (6.25 × 10^5^ BJ-5ta cells in 10 µL) were seeded onto each disc, with incubation for 90 min to allow for attachment. Then, BJ/5ta media (1 mL) was gently added to each well and the cells were incubated on the scaffolds at 37 °C in 5% CO_2_. After 24 h, the cell-laden scaffolds were moved to fresh wells of a 24-well plate with the media replenished. The conditioned medium from the first 24 h of culture was collected, cell debris removed by centrifugation—with storage at −20 °C—prior to the analysis of cell death (as described below). The cell-laden scaffolds were cultured for a further two or seven days, with daily replenishment of media, prior to analysis of cell attachment and morphology (as described below). Three independent experiments, each with three replicate scaffolds, were conducted for each seaweed species and time point.

### 2.11. Measurement of Cytotoxicity

Lactate dehydrogenase (LDH), a cellular enzyme released during cell death, was measured in conditioned medium collected from the scaffolds after 24 h of culture [46]. Samples were also collected from live and dead controls, where 6.25 *×* 10^5^ BJ/5Ta cells (equal to that seeded onto each disc) were seeded into a well of a 24-well plate, with conditioned media collected after 24 h of incubation (live control). Media were then replaced and the cells subjected to three freeze–thaw cycles, with each cycle involving 1 h at −80 °C and 1 h at 65 °C on a hot plate, after which the cell lysate was collected (dead control).

Activity of LDH within the conditioned medium (scaffold, live, and dead controls) was assessed according to the protocol described by Kaja et al. (2015). Briefly, 50 µL of conditioned media was incubated with assay reagent (50 µL) for 60 min, before 1M acetic acid (Merck, 50 µL) was added to stop the reaction, and absorbance measurements were taken at 490 nm [46]. Release of LDH was calculated by subtracting the absorbance for the live control from that of the scaffold and dead control samples:

$$LDH\;release=absorbance\left(sample\;\right)-absorbance\;(live\;control)\;$$

Scaffold-induced cytotoxicity was then calculated as the percentage of the LDH activity for the dead control:

$$Cytotoxicity\;\left(\%\right)=\frac{LDH\;release\;(scaffold)\;}{LDH\;release\;(dead\;control)\;}\times 100$$

### 2.12. Visualization of Cells on Scaffolds

Cell attachment, growth, and distribution on scaffolds were observed at two and seven days post-seeding. Scaffolds were removed from the culture medium, then washed with warm PBS. Cell membrane glycans and cellulose fibers within the scaffold were stained with WGA–AlexaFluor^TM^ 594 conjugate and CW stain, respectively. Briefly, cell scaffolds were fixed with 4% paraformaldehyde (1 mL) for 15 min at room temperature, washed with PBS, then 5.0 μg/mL of WGA (in PBS, 1 mL) was added, prior to incubation overnight at 4 °C. The cell scaffolds were then washed with PBS, followed by incubated in CW stain (1 mL, undiluted) for 1 h at room temperature. Finally, the cell scaffolds were washed with PBS, then placed in PBS in glass-bottom 24-well plates (Greiner Bio-One, Monroe, NC, USA).

Fixed and stained cell scaffolds were imaged (CW, excitation/emission 380/475 nm; WGA, excitation/emission 587/603 nm) using the Andor Dragonfly Spinning Disk Microscopy System (Oxford Instruments, Abingdon, UK). Confocal Z-stacks were taken of the entire scaffold, using both the blue and red channels, with a z-interval of 2 µm. Three independent replicates were imaged for each macroalgae scaffold and time point.

The morphology of cells within scaffolds was examined further through SEM analyses, after confocal analysis, as described previously.

### 2.13. Statistical Analyses

Cross-species comparisons between macroalgae samples and scaffolds were conducted using the non-parametric Kruskal–Wallis test as the sample size was insufficient to assess whether the data followed a normal distribution. Differences of *p* ≤ 0.05 were considered statistically significant.

## 3. Results

### 3.1. Macroalgae Differ in Morphology

**Figure 1:**
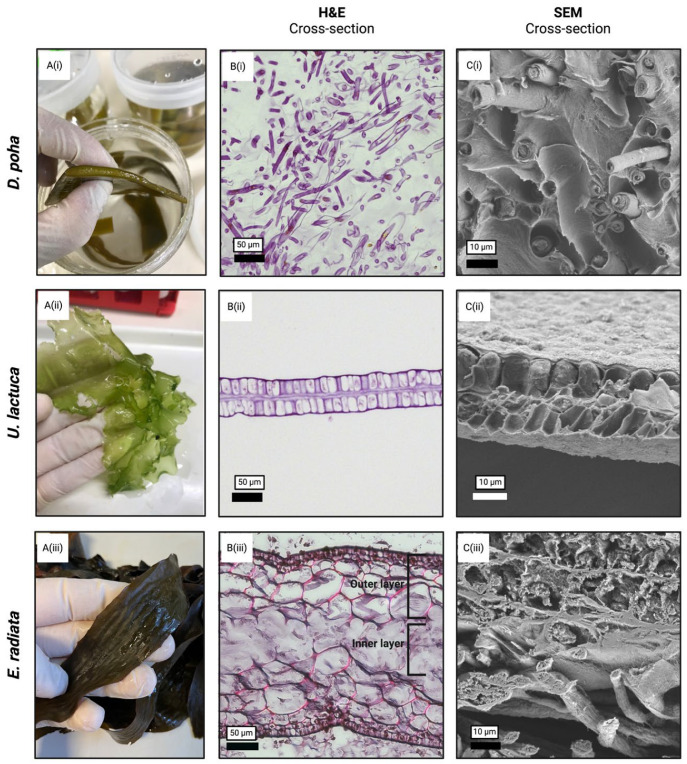
Morphological differences between macroalgae: (**A**) macroscopic images of *Durvillaea poha* (i), *Ulva lactuca* (ii), and *Ecklonia radiata* (iii) illustrate differences in size and morphology; (**B**) H&E staining of paraffin-embedded sections; and (**C**) SEM imaging reveals their porous versus fibrous structural composition. Scale bars are as indicated.

Brown and green seaweeds were obtained and structural features examined using histological and SEM analyses. In its native form, the blade of *D. poha* was thick and flexible with a distinctive, interior medulla (Figure 1A(i)). Microscopic analyses revealed porous cavities with interwoven fibrous hyphae (Figure 1B(i),C(i)). By comparison, the thallus of *U. lactuca* was a thin, near-translucent leafy material (Figure 1A(ii)), with microscopic analysis showing two porous layers of cortex cells (Figure 1B(ii),C(ii)). The secondary lamina of *E. radiata* was thinner than *D. poha* (Figure 1A(iii)) and contained a complex tri-layered structure when observed microscopically (Figure 1B(iii),C(iii)). The outer, porous cortex layers encapsulated the inner, fibrous, medullar layer.

### 3.2. Macroalgae Differ in Extent of Decellularization

Decellularization protocols were first optimized for the blade of *D. poha* due to its increased scale and density in comparison to *U. lactuca* and *E. radiata.* Methods were trialed with increasing chemical intensity, concentration, temperature, and time to identify the minimum intervention required to achieve pigment clearance. Treatments with 0.1% or 0.5% SDS were unsuccessful at removing pigment over the course of 8 days (Supplementary Materials: Figure S1, Table S2), while treatment with 3% NaCO_3_/0.3% NaClO at 60 °C resulted in substantial loss of structural integrity after 24 h. Treatment with 10% SDS reduced pigment by 53.6% after 5 days, with subsequent treatment with 1% Triton X-100/0.1% NaClO achieving pigment clearances of 76.6% after 24 h, and 87.1% once visually cleared (Supplementary Materials: Figure S1, Table S2). Similar levels of pigment clearance were achieved with samples from the *D. poha* palm and stipe (Supplementary Materials: Table S2), although these samples showed greater structural loss with chemical treatment relative to the blade. Further analyses were therefore limited to *D. poha* blade samples.

Once visually cleared, the treated *D. poha* sample had reduced in DNA content by 76.2% relative to the native blade (Supplementary Materials: Table S3). Extension of the 1% Triton X-100/2% NaClO treatment time to 14 days resulted in 97.8% of the DNA content being cleared (Supplementary Materials: Table S3).

The treatment protocol developed for *D. poha* was then tested on samples of *U. lactuca* thallus and *E. radiata* secondary lamina. Pigment loss was observed after incubation in 10% SDS for 5 days, with full clearance achieved with subsequent treatment with 1% Triton X-100/2% NaClO for 14 days (Supplementary Materials: Figure S2). However, the samples had lost structural integrity and were too difficult to handle. Treatment in 1% Triton X-100/0.1% NaClO, omitting the SDS incubation step, resulted in comparable pigment clearance to the two-step protocol while retaining the robust structures of native *U. lactuca* and *E. radiata* (Supplementary Materials: Figure S2). Further, the DNA content of *U. lactuca* and *E. radiata* samples reduced with treatment time, reaching 81.6% and 93.7% DNA clearance, respectively, after 14 days incubation (Supplementary Materials: Table S3).

The two-step decellularization protocol for *D. poha* achieved 87.1 ± 1.9% clearance of pigment relative to the native material (Table 1). The single-step protocol for *U. lactuca* and *E. radiata* achieved similar pigment clearances of 93.1 ± 3.1% and 95.2 ± 1.3%, respectively (*p* > 0.05). Pigment clearance did not correlate with the pigment content in native samples, which was significantly higher in the brown species than *U. lactuca* (*p* ≤ 0.05). The extent of DNA clearance with chemical treatment, however, varied with macroalgae species and inversely correlated with the DNA content of the native samples. The native *U. lactuca* had a greater DNA content (96.7 ± 21.8 ng/mg) than the brown species (*p* ≤ 0.05), with a 69.9 ± 7.7% post-treatment reduction. By contrast, significantly greater DNA clearance of 97.7 ± 0.6% was achieved with *D. poha* treatment (*p* ≤ 0.05), which had the least DNA content of the native samples (11.7 ± 2.6 ng/mg). Native *E. radiata* had an intermediate DNA content (33.4 ± 3.8 ng/mg), with treatment resulting in 82.9 ± 5.8% clearance of DNA.

### 3.3. Macroalgae Matrices Retain Structural Differences

**Figure 2:**
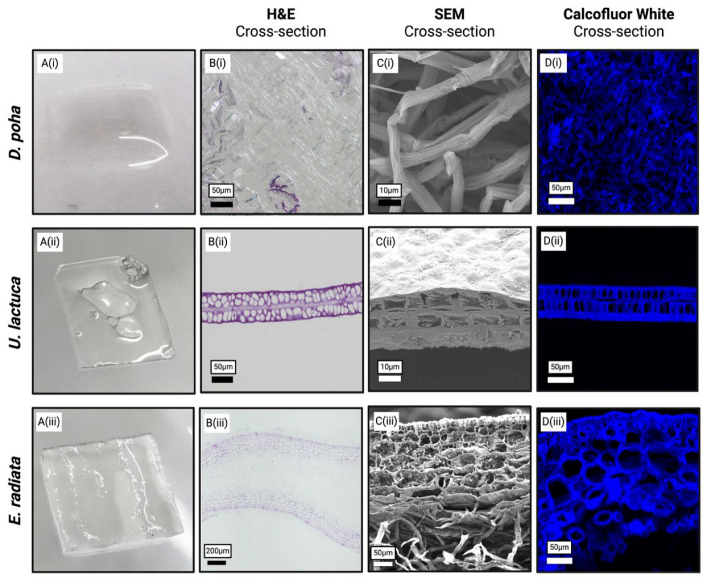
Structural differences between macroalgae matrices: (**A**) Images of *Durvillaea poha* (i), *Ulva lactuca* (ii), and *Ecklonia radiata* (iii) matrices demonstrate macrostructure after chemical treatment. (**B**) H&E staining of paraffin-embedded sections, (**C**) SEM imaging, and (**D**) calcofluor white staining of paraffin-embedded sections show porous and/or fibrous compositions. Scale bars are as indicated.

Structural features of the decellularized macroalgae samples were assessed using SEM and histological analyses. Chemical treatment of *D. poha* resulted in a soft hydrogel (Figure 2A(i)), while the *U. lactuca* and *E. radiata* formed thin films (Figure 2A(ii,iii)). The *D. poha* matrix was composed of interconnecting fibers that lacked the porous structure of native tissue (Figure 2B(i),C(i)). The *U. lactuca* matrix, however, retained the native cortex layer (Figure 2B(ii),C(ii)). Similarly, the *E. radiata* matrix was composed of outer porous and inner fibrous layers (Figure 2B(iii),C(iii)) that swelled relative to the native structure. No nuclear material could be visualized after chemical treatment in any matrix but differences were observed in the staining of other tissue components (Figure 2B(i–iii)). However, cellulose staining was evident in the porous and/or fibrous layers of each matrix (Figure 2D(i–iii)).

### 3.4. Macroalgae Matrices Differ in Physical Properties

Porosity of the macroalgae matrices was measured to assess suitability for use as cellular scaffolds (Table 2). Gaps between fibers were 3.5 times greater within the *E. radiata* inner layer than for *D. poha* (*p* ≤ 0.05). Pores within the outer *E. radiata* layers were also 3.2 times greater in diameter than those of *U. lactuca* (*p* ≤ 0.05). Hydration and stability of the macroalgae matrices were also measured under cell culture conditions (Table 2). Swelling ratios were similar for all matrices at 24 h (*p* > 0.05), with 33- to 38-fold increases in weight. After seven days of culture, there were no significant differences in scaffold stability (*p* > 0.05), although the *E. radiata* samples had a mass loss of 19.5 ± 2.4% relative to the 35–36% losses for *U. lactuca* and *D. poha*.

### 3.5. Macroalgae Matrices Differ in Suitability as Cell Scaffolds

Cytotoxicity of the macroalgae matrices was assessed after 24 h of culture with human dermal fibroblasts through the measurement of LDH release. All matrices were tolerated by the cells, irrespective of macroalgae source, with cytotoxicity levels substantially below that of the dead control (Supplementary Materials: Figure S3). However, the level of cell death detected with *E. radiata* scaffolds (7.9 ± 1.7%) was significantly greater than that for *D. poha* scaffolds (2.3 ± 0.9%, *p* ≤ 0.05) but not *U. lactuca* scaffolds (5.0 ± 1.1%).

**Figure 3:**
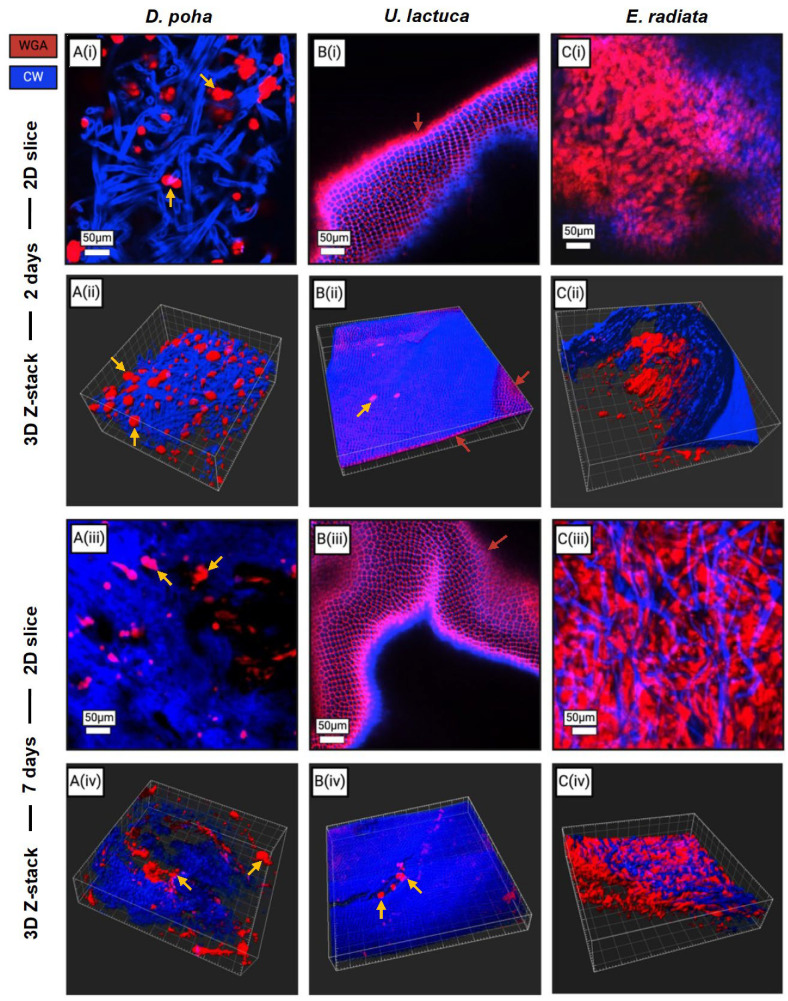
Human dermal fibroblasts attach to and distribute through the fibrous layers of *Ecklonia radiata* but not *Ulva lactuca* and *Durvillaea poha* scaffolds. Representative two-dimensional optical slices (i,iii) and three-dimensional confocal Z-stacks (ii,iv) showing BJ/5Ta cells cultured on *Durvillaea poha* (**A**), *Ulva lactuca* (**B**), and *Ecklonia radiata* (**C**) matrices for two (i,ii) or seven (iii,iv) days, after fixation and staining with wheat germ agglutinin (WGA; red) and calcofluor white (CW; blue) to visualize cell membrane glycans and cellulose fibers, respectively. Gold arrows show cells and spheroids that have failed to attach to cellulose fibers. Red arrows show acellular punctate staining. Scale bars are as indicated.

Distribution of fibroblasts within the macroalgae matrices was examined at two and seven days post-seeding, with cellulose and cell membrane stains visualized by confocal microscopy (Figure 3). Within the *D. poha* scaffolds, rounded cells and spheroids were observed that had failed to attach to the cellulose fibers and decreased in size and number over the culture period (Figure 3A(i–iv)). Punctate, red staining was detected on *U. lactuca* scaffolds (Figure 3B(i–iii)). Binding of WGA to the matrix surface was also observed in the absence of cells, as shown in paraffin-embedded sections (Supplementary Materials: Figure S4). Due to this matrix staining, cells were difficult to visualize on the *U. lactuca* scaffolds but those that were identified had failed to attach to the cellulose or increase in number over time (Figure 3B(ii,iv)). By contrast, in the *E. radiata* scaffolds, cells were closely associated with the cellulose, with a spreading morphology indicative of attachment (Figure 3C(i–iv)). Further, the cells were limited to the inner layer of cellulose fibers at day 2 (Figure 3C(ii)), with increased distribution throughout the scaffold by day 7 (Figure 3C(iv)).

**Figure 4:**
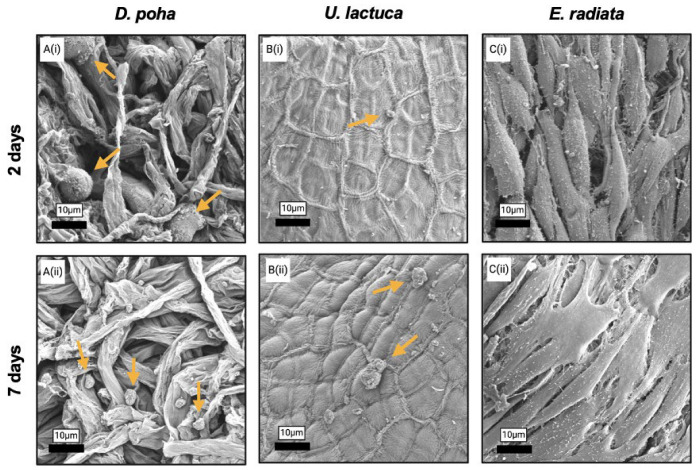
Fibroblasts differ in their attachment to and morphology on macroalgae scaffolds. Scanning electron microscopy images showing BJ/5Ta cells cultured on *Durvillaea poha* (**A**), *Ulva lactuca* (**B**), and *Ecklonia radiata* (**C**) matrices for two (i) or seven (ii) days. Gold arrows show cells and spheroids lacking fibroblastic morphology. Scale bars are as indicated.

Fibroblast attachment to and growth within the macroalgae matrices were also assessed by SEM (Figure 4). As observed by confocal microscopy, rounded cells and spheroids were located between the *D. poha* fibers, with a decreased size noted over time (Figure 4A(i,ii)). Rounded cells were visualized in limited numbers on the *U. lactuca* surface, with no changes in morphology evident from day 2 to day 7 (Figure 4B(i,ii)). Previous observations of the *E. radiata* scaffolds were confirmed by SEM imaging, with cell spreading and flattening, consistent with a fibroblastic morphology and increasing with culture time (Figure 4C(i,ii)).

## 4. Discussion

Three distinct macroalgae were decellularized and the resulting cellulose-based matrices evaluated as scaffolds for dermal tissue engineering. Decellularization protocols were tailored for each species, achieving substantial pigment and varied DNA clearance. Cellulose matrices generated from *U. lactuca* and *E. radiata* retained the native ECM structure but this was not the case for the *D. poha* matrix. While the matrices showed equivalent hydrophilicity and stability under culture conditions, species-specific differences in porosity were observed, with *E. radiata* samples providing the most open structure. The cellulose matrices showed minimal toxicity for human dermal fibroblasts but substantial differences were observed in their capacity to support cell attachment and spreading. When seeded on *D. poha* or *U. lactuca* scaffolds, fibroblasts were rounded or formed spheroids with limited cellulose contact. By contrast, fibroblasts attached to the fibrous inner layer of the *E. radiata* scaffolds, with morphological changes exhibited over the culture period.

Macroalgae were decellularized using one- or two-step protocols involving the use of surfactants and bleach without thermal treatment, as previously applied to terrestrial plants [31,43] and recently to green and red species of macroalgae [47]. The protocols used in this study contrast with the four-step protocol applied previously to *Ulva* sp. and *Cladophora* sp. [41], which utilized solvent, bleach, alkali treatment, and acid hydrolysis at temperatures from 60 to 100 °C. The protocols were more consistent with the one-step SDS (1%) or Triton-X-100 (1%), room-temperature treatments recently reported for *U. lactuca* and *Rhodophyta* [47]. A point of difference between this and the prior studies was storage conditions prior to microalgae treatment. Whereas other studies used fresh material, here, *E. radiata* was dried then rehydrated while *U. lactuca* and *D. poha* were frozen then defrosted prior to treatment. These steps may have aided decellularization as osmotic shock and freeze-thawing have been shown to aid in the extraction of macroalgae protein [48,49]. The protocols developed removed 87–95% of macroalgae pigments, with a greater variation in DNA clearance from 70 to 98%. This inversely correlated with DNA content in the native tissue, which varied from 12 ng/mg for *D. poha* to 97 ng/mg for *U. lactuca*.

The native DNA content was consistent with previous reports for brown macroalgae species [50] but greater than that for *Ulva* sp. [41], and likely reflected the ratio of cortex to medulla in the samples used. Further, the residual DNA content after decellularization was similar to that reported for green macroalgae and terrestrial plants that were successfully recellularized in vitro [33,41,43]. Residual DNA may not be problematic for in vitro tissue engineering, as bovine skeletal muscle cells were successfully cultured on a matrix generated from the homogenized medulla of the brown macroalgae *Undaria pinnatifida*, despite no attempt at DNA removal [51]. However, standards have been proposed to minimize adverse responses to scaffold implantation as residual DNA in animal-derived scaffolds has been linked to inflammatory reactions [52], which in some cases was fatal [53]. It has been proposed that DNA content within matrices should be less than 50 ng/mg and 200 bp in length, with no nuclear material visible in stained sections [6]. The three macroalgae matrices generated here meet the first and last standard, however, fragment length was not assessed. If DNA content within the macroalgae-derived cellulose matrices causes concern, levels could be reduced further through DNase digestion, as demonstrated with cellulose matrices generated from tobacco hairy root [54]. Mechanical decellularization methods, such as high hydrostatic pressure, supercritical CO_2_, and electroporation [6,11,55,56,57,58], could also be trialed to further reduce chemical usage.

Decellularization protocols employed in this study were aimed at retaining the varied cellulose ECM structures of the macroalgae species chosen. All matrices contained cellulose but varied in retention of native porous and fibrous structures. Native *E. radiata* ECM was the most complex in structure, with outer layers of cortex cells and an inner layer of medulla cells [59], which swelled during decellularization to create a multilayered matrix with both cellulose-lined pores and cellulose fibers. This matrix was hydrophilic and stable under cell culture conditions. Decellularization resulted in the loss of the *D. poha* medulla macrostructure, leaving cellulose fibers likely originating from the long filamentous cells that facilitate nutrient transfer from lamina to holdfast [60]. The *D. poha* matrix was less dense than the native structure, with smaller gaps between fibers than for the inner *E. radiata* matrix, with some degradation observed during culture. Collapse of the *D. poha* matrix may relate to processing conditions such as freeze/thawing or treatment with SDS [60,61,62] and/or the loss of air pockets that provide buoyancy [63] or other structural ECM components such as alginate [64,65]. The *U. lactuca* matrix most resembled the native ECM structure [66], with two tight cortex-derived layers of cellulose-bound pores remaining after decellularization. This contrasted with the decellularized matrix from *Ulva* sp. previously reported [41], which showed a greater separation of the cortex layers with intense chemical and heat treatment. The *U. lactuca* matrix had the smallest pores and greatest eosin and WGA staining of the matrices evaluated here. The plant-derived lectin, WGA, binds cell surface proteoglycans through N-acetylneuraminic (sialic) acid and *N*-acetylgalactosamine [44,67]. Highly glycosylated proteins have been identified in the cell wall and vesicles of *Ulva* sp. [68,69,70], which may account for the staining observed. It may, therefore, be beneficial to compare macroalgae matrix compositions [47,49,71,72] after applying decellularization protocols of varying intensity to assess whether residual components, beyond cellulose, are beneficial or detrimental for use as scaffolds. It would also be beneficial to assess the stability of these matrices under more challenging environments that better mimic human tissues. Although humans lack the enzyme capable of cellulose degradation [73], other scaffold constituents may be susceptible to ionic dissolution, or chemical or enzymatic cleavage [45], which may translate to faster breakdown if utilized in vivo. The impact of microbial cellulases on scaffold stability, as a result of infection or contamination, should also be considered [74,75].

Fibroblasts were seeded on the macroalgae-derived matrices to assess their suitability for dermal tissue engineering. While the *E. radiata* cellulose matrix supported fibroblast attachment, growth, and changes in morphology consistent with their maturation [76], the cells failed to interact with the matrices from *U. lactuca* and *D. poha*. This did not appear to relate to toxicity as the greatest LDH activity indicative of cell lysis was observed with the *E. radiata* scaffold. This initial toxicity may relate to surfactant or bleach retained within the superficial, cortical layer of the *E. radiata* scaffold, which in turn may have limited viable fibroblasts to its fibrous layer. Physical or compositional differences between and within matrices may also have impacted the ability to support fibroblast culture.

The physical impact of cellulose ECM structure on cell fate was not clear, with fibroblasts attaching to cellulose fibers in *E. radiata* scaffolds but not those in *D. poha*. This is in part consistent with the findings from Bar-Shai et al. (2021), who showed fibroblast attachment and maturation on fibrous cellulose matrices from *Cladophora* sp. [41]. Matrix porosity may have influenced outcomes here as the gaps between cellulose fibers were greater in scaffolds from *E. radiata* than *D. poha*, which is consistent with the pore size range deemed suitable for fibroblasts (40–150 µm) [77]. Matrix stiffness may also have played a role [78,79], with the surrounding porous layers of cellulose in *E. radiata* providing strength to the internal fibrous layer, whereas the *D. poha* matrix was composed entirely of unsupported fibers. Measurements of tensile strength would be needed to support this [33] but the *D. poha* scaffold was softer and more fragile to handle than that of *E. radiata*. Cellulose fiber topography may have also differed between the *E. radiata* and *D. poha* matrices [80], which could account for the differences in the fibroblast adhesion, distribution, and spreading morphology observed [81,82].

Polysaccharides within the ECM of human tissues provide mechanical signals to cell surface receptors, such as integrins, which enable cells to respond to their physical environment [83]. Thus, differences in composition between the *E. radiata* and *D. poha* scaffolds may also have affected the cellular outcomes. Indeed, polysaccharides found in brown macroalgae reportedly have differential effects on fibroblast activity. Fucoidan has been shown to promote fibroblast proliferation and influence integrin and ECM production [84,85], while alginate generally requires modifications to facilitate fibroblast attachment [86]. The *U. lactuca* matrices generated here, like those from *D. poha*, also failed to support cell attachment, which contrasts with prior reports [41,47], although the microscopic images provided by these studies indicate that keratinocyte growth on the matrix surface was superior to that of fibroblasts. Future studies could compare the impact of decellularization protocols on matrix porosity, stiffness, topography, and composition to determine the scaffold parameters most supportive of different skin cell cultures. Matrices that fail to support cell culture may still have utility if protein-coated to improve cell attachment [29,33,87,88] or loaded with drugs as delivery systems [47,89].

In summary, this study highlighted the influence of the native ECM structure of macroalgae on decellularization efficiency, cellulose-based matrix properties, and scaffold utility. A macroalgae species and low-intensity, decellularization protocol was successfully identified that generated a cellulose-based matrix compatible with human dermal fibroblast culture. Future studies can explore the potential of this multilayered, *E. radiata* scaffold for skin tissue engineering, for example, through simultaneous culture of keratinocytes, fibroblasts, and vascular endothelial cells in vitro and in vivo application to skin wounds [90,91,92]. The primary limitation of this study was that the decellularized matrices generated from *D. poha* and *U. lactuca* failed as scaffolds for skin tissue engineering. This suggests that specific microalgae species and/or tailored decellularization protocols may be needed to refine the chemical and physical properties of cellulose matrices to produce scaffolds compatible with cells and tissues of interest. Following this refinement, matrices generated from diverse macroalgae species may find utility in a range of tissue engineering applications.

## Figures and Tables

**Table 1:** Macroalgae vary in DNA but not pigment clearance after chemical treatment.

Species	Native Pigment Content (MGV) *	Pigment Clearance (%) *^†^	Native DNA Content (ng/mg) *	DNA Clearance (%) *^†^
*D. poha*	167.2 ± 3.2 ^a^	87.1 ± 1.9	11.7 ± 2.6 ^a^	97.7 ± 0.6 ^a^
*U. lactuca*	109.2 ± 2.8 ^a,b^	93.1 ± 3.1	96.7 ± 21.8 ^a,b^	69.9 ± 7.7 ^a^
*E. radiata*	157.0 ± 0.5 ^b^	95.2 ± 1.3	33.4 ± 3.8 ^b^	82.9 ± 5.8

* Mean ± SEM; n = 3. ^†^ Percentage reduction relative to native seaweed. MGV: mean gray value. Means that differ significantly across species share superscript letters (*p* ≤ 0.05).

**Table 2:** Physical differences between decellularized macroalgae matrices.

Species	Pore Diameter (µm) *	Swelling Ratio (FC) *^†^	Mass Loss (%) *^#^
*D. poha*	23.9 ± 2.07 ^a^	33.4 ± 6.9	35.5 ± 5.4
*U. lactuca*	6.6 ± 0.9 ^b^	39.2 ± 5.1	36.1 ± 6.0
*E. radiata*	82.7 ± 9.0 (inner) ^a,b,c^ 21.4 ± 2.0 (outer) ^b,c^	34.4 ± 5.9	19.52 ± 2.4

* Mean ± SEM; n = 3. ^†^ Fold change (FC) in wet weight at 24 h relative to dry weight at day 0. ^#^ Percentage reduction in wet weight at day 7 relative to wet weight at day 0. Means that differ significantly across species share superscript letters (*p* ≤ 0.05).

## Data Availability

The original contributions presented in the study are included in the article/Supplementary Materials, further inquiries can be directed to the corresponding author.

## Supplementary Materials

The following supporting information can be downloaded at https://www.mdpi.com/article/10.3390/jfb15120390/s1, Figure S1: Treatment parameters trialed in the decellularization of *Durvillaea poha*; Figure S2: Treatment parameters trialed in the decellularization of *Ulva lactuca* and *Ecklonia radiata*; Figure S3: Macroalgae matrices are biocompatible with human dermal fibroblasts; Figure S4: Wheat germ agglutinin stains glycans on the surface of *Ulva lactuca* scaffolds in the absence of cells; Table S1: Parameters trialed in macroalgae decellularization protocols; Table S2: Pigment clearance from *Durvillaea poha* differs with chemical treatment; Table S3: DNA clearance from macroalgae species differs with chemical treatment.
