# Supplementary material for: Decellularized Green and Brown Macroalgae as Cellulose Matrices for Tissue Engineering
Source: J Funct Biomater. 2024 Dec 23;15(12):390. doi: 10.3390/jfb15120390 (PMC11677820; doi:10.3390/jfb15120390)
Supplement: Supplementary file 1 [file jfb-15-00390-s001.zip › jfb-3344548-supplementary.pdf]

Supplementary Materials:

Table S1. *Parameters trialled in macroalgae decellularization protocols*

	Chemical treatment		
**Parameter**	SDS	$NaCO_2$ / NaClO	Triton X-100 / NaClO
Concentration (%)	0.1, 0.5, 10	3 / 0.3	1 / 0.1
Time (days)	0.5, 2, 5, 6, 8	1	1, 14, until cleared
Temp (°C)	20	60	20, 60

Table S2. *Pigment clearance from Durvillaea poha differs with chemical treatment*

	Pigment clearance (%)*		
**Treatment protocol**	**Blade**	**Palm**	**Stipe**
0.1% SDS; 20ºC; 2 days[#]	18.4	18.8	19.4
0.1% SDS; 20ºC; 8 days[#]	17.3	14.4	-9.5
0.5% SDS; 20ºC; 0.5 days - extended to 6 days[†]	23.4	17.0	4.2
3% $NaCO_3$ / 0.3% NaClO; 60ºC; 1 day[§]	25.8	-34.6	-64.3
10% SDS; 20ºC; 5 days; followed by 1% Triton X-100 / 0.1% NaClO; 60ºC; 1 day[§]	76.6	61.2	25.0
10% SDS; 20ºC; 5 days; followed by 1% Triton X-100/0.1% NaClO; 20ºC; until cleared[§]	87.1	89.3	86.4

** Pigment clearance is presented as the mean percentage reduction in mean gray value relative to native; n ≤ 3. Adapted from [#]Modulevsky et al., 2016, [†]Modulevsky et al., 2014, or [§]Adamski et al., 2018.*

Table S3. *DNA clearance from macroalgae species differs with chemical treatment*

	DNA clearance (%)*		
**Treatment Protocol**	***D. poha***	***U. lactuca***	***E. radiata***
10% SDS; 20ºC; 5 days; followed by 1% Triton X-100 / 0.1% NaClO; 20ºC; until cleared	76.2	-	-
10% SDS; 20ºC; 5 days; followed by 1% Triton X-100 / 0.1% NaClO; 20ºC; 14 days	97.8	-	-
1% Triton X-100 / 0.1% NaClO; 20ºC; 5 days	-	84.0	64.5
1% Triton X-100 / 0.1% NaClO; 20ºC; 7 days	-	81.5	78.0
1% Triton X-100 / 0.1% NaClO; 20ºC; 10 days	-	69.3	80.3
1% Triton X-100 / 0.1% NaClO; 20ºC; 14 days	-	81.6	93.7

** DNA clearance is presented as the percentage reduction in ng DNA/mg tissue relative to native; n = 1.*

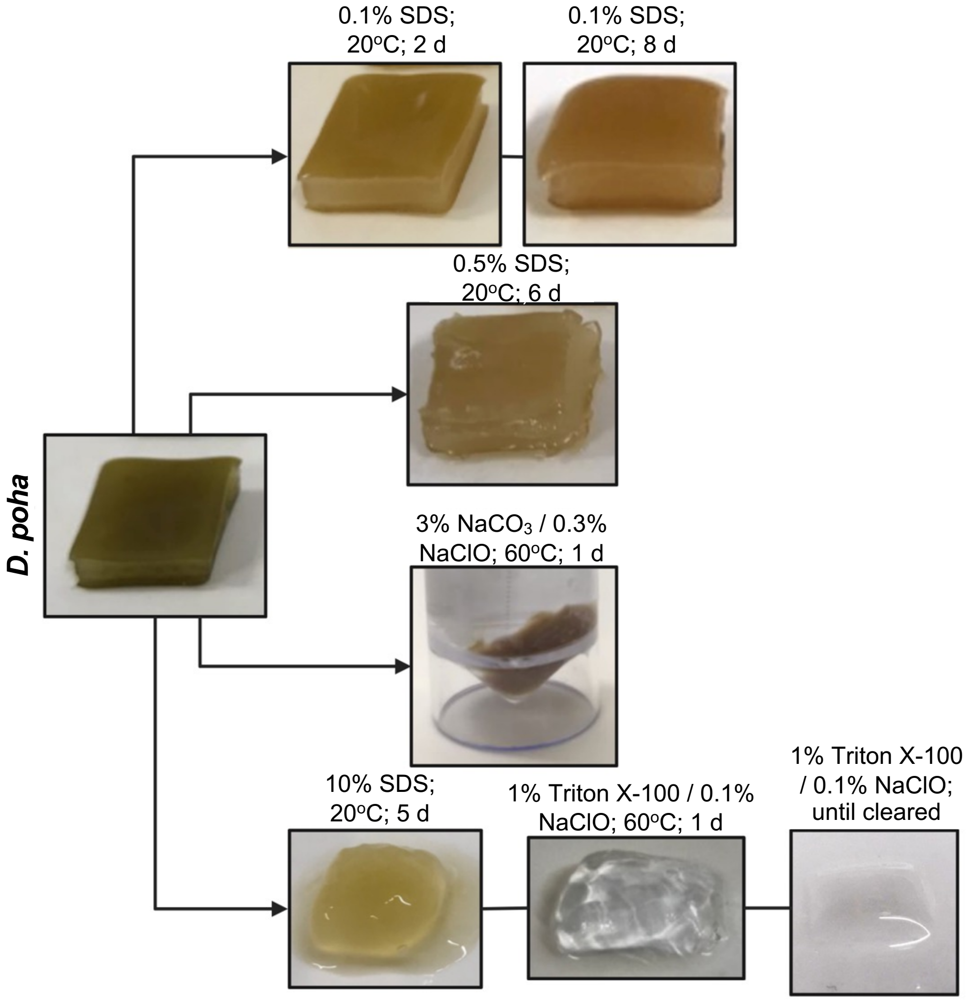

Figure S1. *Treatment parameters trialled in the decellularization of Durvillaea poha. Macroscopic images of D. poha blade samples illustrate differences in their structural integrity and pigment clearance relative to chemical treatment.*

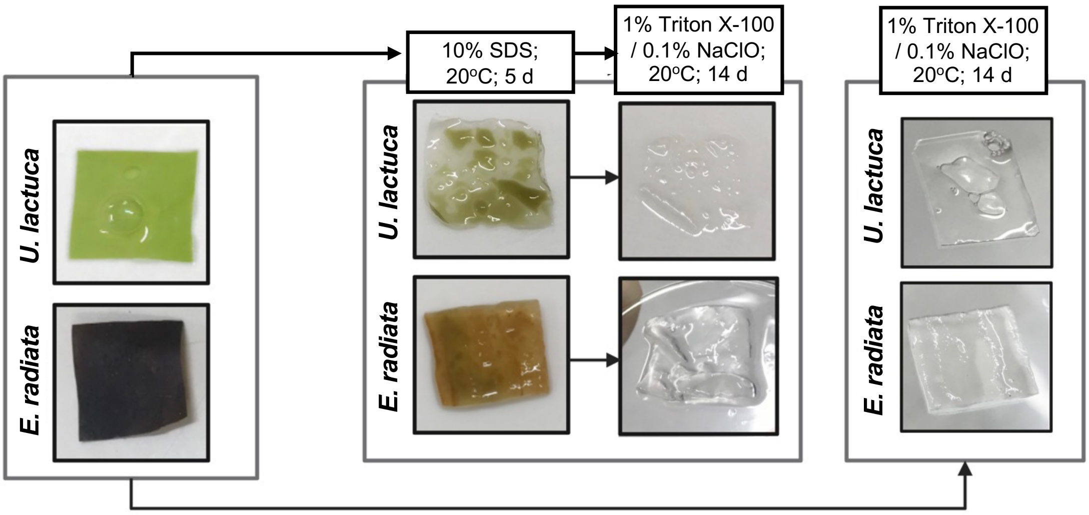

Figure S2. *Treatment parameters trialled in the decellularization of Ulva lactuca and Ecklonia radiata. Macroscopic images of macroalgae samples illustrate differences in their structural integrity and pigment clearance relative to chemical treatment.*

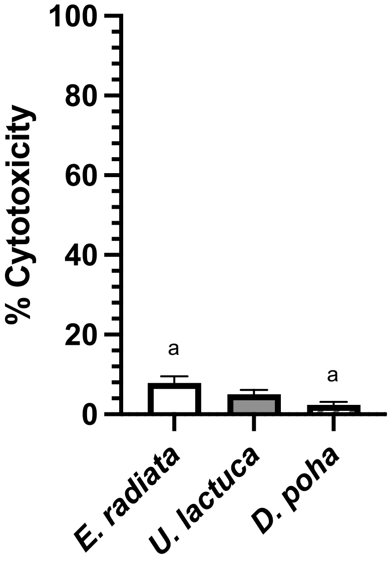

Figure S3. *Macroalgae matrices are biocompatible with human dermal fibroblasts. Durvillaea poha, Ulva lactuca and Ecklonia radiata matrices were seeded with BJ/5Ta cells, with lactate dehydrogenase activity in the conditioned media measured after 24 hours. Data is presented as the mean percentage ± SEM relative to a cytotoxic control; n = 3. Means that differ significantly share letters (p ≤ 0.05).*

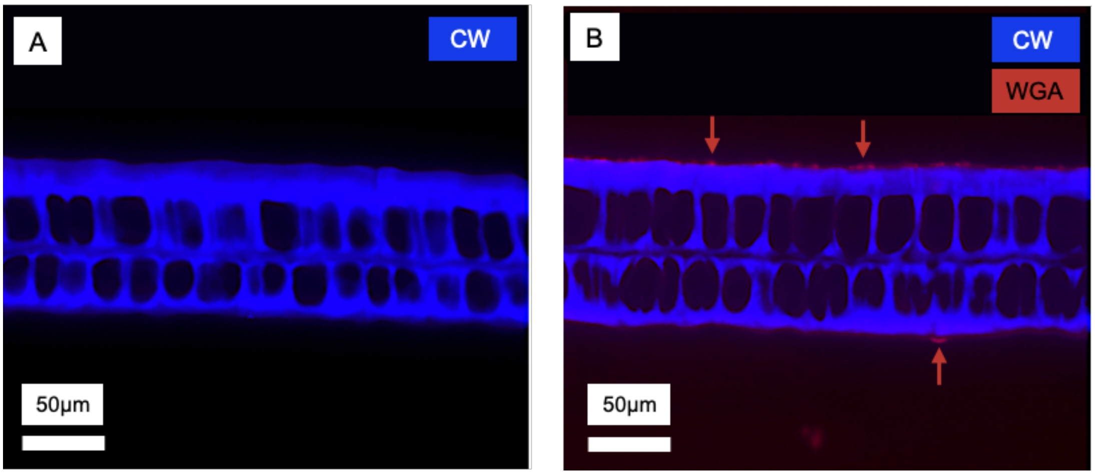

Figure S4. *Wheat germ agglutinin stains glycans on the surface of Ulva lactuca scaffolds in the absence of cells. Representative images of fixed and paraffin-embedded sections of U. lactuca scaffolds after staining with (**A**) calcofluor white (CW; blue) alone or (**B**) in combination with wheat germ agglutinin (WGA; red). Red arrows show surface glycans. Scale bars are as indicated.*
